# Evaluation of zinc sulfate as an adjunctive therapy in COVID-19 critically ill patients: a two center propensity-score matched study

**DOI:** 10.1186/s13054-021-03785-1

**Published:** 2021-10-18

**Authors:** Khalid Al Sulaiman, Ohoud Aljuhani, Abdulrahman I. Al Shaya, Abdullah Kharbosh, Raed Kensara, Alhomaidi Al Guwairy, Aisha Alharbi, Rahmah Algarni, Shmeylan Al Harbi, Ramesh Vishwakarma, Ghazwa B. Korayem

**Affiliations:** 1 Pharmaceutical Care Department, King Abdulaziz Medical City, Riyadh, Saudi Arabia; 2 King Abdullah International Medical Research Center, Riyadh, Saudi Arabia; 3 Department of Pharmacy Practice, Faculty of Pharmacy, King Abdulaziz University, Jeddah, Saudi Arabia; 4 College of Pharmacy, King Saud Bin Abdulaziz University for Health Sciences, Riyadh, Saudi Arabia; 5 Department of Clinical Pharmacy, Faculty of Pharmacy, Taif University, Taif, Saudi Arabia; 6 Pharmaceutical Care Department, King Abdulaziz University Hospital, Jeddah, Saudi Arabia; 7 Department of Pharmacy Practice, College of Pharmacy, Princess Nourah Bint Abdulrahman University, Riyadh, Saudi Arabia

**Keywords:** COVID-19, SARS-CoV-2, Zinc, Supplements, Trace element, Critically ill, Intensive care unit (ICU), 30-Day mortality

## Abstract

**Background:**

Zinc is a trace element that plays a role in stimulating innate and acquired immunity. The role of zinc in critically ill patients with COVID-19 remains unclear. This study aims to evaluate the efficacy and safety of zinc sulfate as adjunctive therapy in critically ill patients with COVID-19.

**Methods:**

Patients aged ≥ 18 years with COVID-19 who were admitted to the intensive care unit (ICU) in two tertiary hospitals in Saudi Arabia were retrospectively assessed for zinc use from March 1, 2020 until March 31, 2021. After propensity score matching (1:1 ratio) based on the selected criteria, we assessed the association of zinc used as adjunctive therapy with the 30-day mortality. Secondary outcomes included the in-hospital mortality, ventilator free days, ICU length of stay (LOS), hospital LOS, and complication (s) during ICU stay.

**Results:**

A total of 164 patients were included, 82 patients received zinc. Patients who received zinc sulfate as adjunctive therapy have a lower 30-day mortality (HR 0.52, CI 0.29, 0.92; *p* = 0.03). On the other hand, the in-hospital mortality was not statistically significant between the two groups (HR 0.64, CI 0.37–1.10; *p* = 0.11). Zinc sulfate use was associated with a lower odds of acute kidney injury development during ICU stay (OR 0.46 CI 0.19–1.06; *p* = 0.07); however, it did not reach statistical significance.

**Conclusion:**

The use of zinc sulfate as an additional treatment in critically ill COVID-19 patients may improve survival. Furthermore, zinc supplementation may have a protective effect on the kidneys.

**Supplementary Information:**

The online version contains supplementary material available at 10.1186/s13054-021-03785-1.

## Background

The coronavirus disease 2019 (COVID-19) caused by the severe acute respiratory syndrome coronavirus 2 (SARS-CoV-2), has emerged as a threat to public health worldwide [1]. Symptoms of COVID-19 manifest as a cluster of mild to severe respiratory symptoms [1]. Severe COVID-19 amplifies the overall systematic inflammatory response in critically ill patients, increasing the risk of multi-organ dysfunctions, acute respiratory disease syndrome (ARDS), and mortality [1–4]. This inflammatory response is caused by the hyperactivation of chemokines and cytokines, prominently interleukin-6 (IL-6) [5]. Therefore, the dysregulation of cytokines was one of the targets for many treatment strategies in treating patients with severe COVID-19 [3].

To date, no specific pharmacological agents have proven efficacy against SARS-CoV; instead, host-directed therapies and supportive therapy are used [5]. The current treatment options for critically ill patients with COVID-19 include anti-viral agents, immunosuppressive agents, and immunomodulators [6]. However, the evidence about these treatment options' mortality benefit in critically ill patients with COVID-19 is conflicting [7–10]. Moreover, some mineral supplements and vitamins with immunomodulatory activity and antioxidant effects such as thiamine, vitamins C and D, zinc, and selenium use in patients with COVID-19 are investigated [11–14]

Zinc is a trace element that plays a role in the development and function of the immune system exhibiting direct or indirect anti-viral properties [13, 14]. High intracellular zinc levels were reported to stall the replication of coronavirus (SARS-CoV-2) and influenza virus that caused the severe acute respiratory syndrome [14–16]. Some reports demonstrate the synergistic effect of zinc with anti-viral therapy in SARS-CoV-2 [17]. The benefit of zinc supplementation on the immune system function has been previously observed in non-COVID-19 patients [17, 18]. However, there is inadequate evidence to support the use of zinc for COVID-19 treatment in critically ill patients [19, 20]. Yet, providers continue to use it as adjunctive therapy in this patient population. Therefore, we aim to evaluate the efficacy and safety of zinc supplementation as adjunctive therapy in treating critically ill patients with COVID-19.

## Methods

### Study design

This was a two-center, retrospective study of all critically ill patients with COVID-19 who were admitted to the Intensive Care Unit (ICU) from March 1, 2020 until March 31, 2021. We aimed to enroll as many patients as possible, with no predefined sample size. The COVID-19 was confirmed using a reverse transcriptase-polymerase chain reaction (RT-PCR) obtained from nasopharyngeal or throat swabs. The study was approved by King Abdullah International Medical Research Center (KAIMRC)-Institutional Review Board, Riyadh, Saudi Arabia (Study Number: NRC21R/287/07).

### Participants

**Fig. 1:**
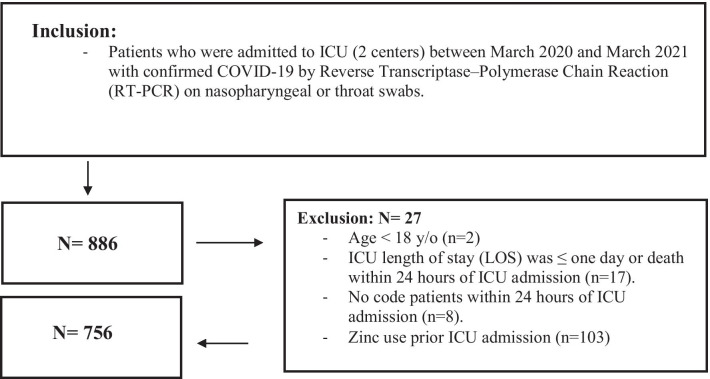
Inclusion/exclusion criteria

Patients aged ≥ 18 years and admitted to ICU for more than 24 h with a confirmed COVID-19 were eligible for inclusion. Patients were excluded if they died within 24 h of ICU admission, labeled as "Do-Not-Resuscitate" within 24 h of ICU admission, or received zinc before ICU admission (Fig. 1). Enrolled patients were classified into two groups based on the administration of zinc sulfate as adjunctive therapy during ICU stay. Patients who received new initiation of zinc sulfate during ICU stay were included in the active group. The decision on whether to use zinc sulfate with a daily dose of 220 mg (50 mg of elemental zinc) enteral tablets for patients was solely based on physician clinical judgment. Patients were followed during their hospital stay, until discharge or in-hospital death, whichever occurred first.

### Setting

This study was conducted in two large, tertiary governmental hospitals; King Abdulaziz Medical City (KAMC), Riyadh, and King Abdulaziz University Hospital (KAUH), Jeddah. The ICUs admit medical, surgical, trauma, burn, and transplant patients and operate as a closed unit with 24/7 onsite coverage by critical care board-certified intensivists and clinical pharmacists. The primary site for this study was King Abdulaziz Medical City.

### Data collection

Each patients’ data were collected and managed using Research Electronic Data Capture (REDCap®) software hosted by King Abdullah International Medical Research Center (KAIMRC). Patient's demographic data, comorbidities, vital signs, and laboratory tests were extracted from electronic medical records. The laboratory tests included the renal profile (Estimated glomerular filtration rate (eGFR), AKI status), liver profile (Aspartate transaminase (AST), Alanine transaminase (ALT), and total bilirubin), coagulation profile (international normalized ratio (INR), activated Partial Thromboplastin Time (aPTT)), and albumin levels within 24 h of ICU admission. In addition, surrogate biomarkers of the COVID-19-associated inflammation (ferritin, D-dimer, fibrinogen and C-reactive protein (CRP)) were collected and reported with their baseline and peak values during the ICU stay [30]. Peak levels were defined to be the highest level of the surrogate marker checked at any time during their ICU length of stay. The aim of investigating these biomarkers was to establish a reasonable correlation between zinc administration and disease progression that might be captured via an increase in the inflammatory surrogate markers. The selected biomarkers have been associated with poor outcomes in COVID-19 patients [4, 30]. The severity baseline scores (i.e., Acute Physiology and Chronic Health Evaluation II (APACHE II), Sequential Organ Failure Assessment (SOFA)), and Nutrition Risk in Critically ill (NUTRIC)) were calculated using the MDCalc website for each patient [20]. In addition, we collected the needs for mechanical ventilation (MV), and MV parameters (e.g., PaO_2_/FiO_2_ ratio, FiO_2_ requirement) within 24 h of ICU admission. Lastly, tocilizumab and corticosteroids use within 24 h and the concomitant use of nephrotoxic medications (i.e. Vancomycin IV, Gentamicin IV, Amikacin IV, Colistin IV, Furosemide, Sulfamethoxazole/trimethoprim and Contrast,) during ICU stay were recorded for the eligible patients.

### Aim of study and outcomes

This study aimed to evaluate the efficacy and safety of zinc sulfate as adjunctive therapy in critically ill patients with COVID-19. The primary endpoint was the 30-day mortality in critically ill patients who received zinc sulfate as adjunctive therapy. The secondary endpoint included the in-hospital mortality, ICU length of stay (LOS), hospital LOS, ventilator free days (VFDs), and evaluation of complications during ICU stay after zinc initiation, including acute kidney injury (AKI), liver injury, and thrombosis/infraction during ICU stay.

### Definition(s)

- The 30-day mortality was defined as the in-hospital death occurring for any cause within 30 days of the admission date during the hospital stay.
- Ventilator-free days (VFDs) at 30 days were calculated as the following: if the patients die within 30 days of MV, the VFDs = 0, VFDs = 30-days after MV initiation (if the patient survived and was successfully liberated from MV), and VFDs = 0 if the patient is on MV for > 30 days.
- Acute kidney injury (AKI) was defined using the Acute Kidney Injury Network (AKIN) definition [21].
- Thrombosis/infraction was defined using the International Statistical Classification of Diseases (ICD) 10-CM code (i.e., Myocardial infarction (MI), ischemic stroke, pulmonary embolism, deep vein thrombosis) [22]
- Respiratory failure was defined as either hypoxemic respiratory failure (PaO_2_ < 60 mm Hg with a normal or low arterial carbon dioxide tension (PaCO_2_) or hypercapnic respiratory failure (PaCO_2_ > 50 mm Hg) that requires invasive mechanical ventilation.
- Liver injury is defined as alanine aminotransferase (ALT) exceeding three times the upper limit of normal or double in patients with elevated baseline ALT.

### Data management and statistical analysis

Categorical variables were reported using numbers and percentages, whereas continuous variables were reported using mean with standard deviation (SD) or median with interquartile range (IQR) when appropriate. We compared categorical variables using the Chi-square or Fisher exact test. Continuous variables were compared using numerical student *t* test (for the normally distributed variables) or other quantitative variables with the Mann–Whitney *U* test (for the non-normally distributed variables). The normality assumptions were assessed for all numerical variables using statistical tests (i.e., Shapiro–Wilk test) and graphical representation (i.e., histograms and Q–Q plots). Model fit assessed using the Hosmer–Lemeshow goodness-of-fit test.

Propensity score matching procedure (Proc PS match) (SAS, Cary, NC) was used to match patients who newly received zinc sulfate (active group) to patients who did not (control group) based on patient’s APACHE II score, acute kidney injury, and systemic use of corticosteroids within 24 h of ICU admission. A greedy nearest neighbor matching method was used in which one patient who newly received zinc sulfate (active) group matched with one patient who did not (control), which eventually produced the smallest within-pair difference among all available pairs with treated patients. Patients were matched only if the difference in the logits of the propensity scores for pairs of patients from the two groups was less than or equal to 0.5 times the pooled estimate of the standard deviation.

Multivariable Cox proportional hazards regression analysis were performed for the 30-day and in-hospital mortality. Additionally, Kaplan–Meier (KM) plots were generated for these outcomes. Multivariable regression analysis and negative binomial regression were used as appropriate for the other outcomes considered in this study. Regression analysis was done by consider PS score as one of the covariates in the model. The odds ratios (OR), hazard ratio (HR), or estimates with the 95% confidence intervals (CI) were reported as appropriate. No imputation was made for missing data as the cohort of patients in our study was not derived from random selection. We considered a* P* value of < 0.05 statistically significant and used SAS version 9.4 for all statistical analysis.

## Results

A total of 756 eligible critically ill patients with COVID-19 were admitted to the ICU during the study period in the two study centers. Zinc sulfate 220 mg (50 mg of elemental zinc) enteral tablets once daily was newly initiated in the ICU to 90 patients, whereas 666 patients did not receive zinc as adjunctive therapy. We matched 164 patients using propensity score (1:1) according to the selected criteria. A total of 38 patients (46.3%) have received zinc sulfate within 24 h of ICU admission. The median (Q1, Q3) duration of zinc sulfate was 11 days (6, 15).

### Study population

Before PS matching, most of the patients in both arms were men (70.9%), and the average age was 60.8 ± 14.6 years in the whole cohort. The most common comorbidities were diabetes mellitus (60.1%), followed by hypertension (56.8%), and dyslipidemia (20.8%) (Additional file 1: Table e1). Moreover, there was a significant difference in the baseline characteristics between the two groups before PS. The baseline severity scores (i.e., APACHE II and SOFA scores), the nutritional status based on the NUTRIC score stratification, total WBCs, procalcitonin levels, AKI status, and INR were higher in the control group. Conversely, patients in the zinc group have a higher eGFR baseline and received more pharmacological deep vein thrombosis prophylaxis. However, after using PS matching based on the selected criteria, most of the baseline characteristics and comorbidities were balanced between the two groups except for lactic acid baseline, which was significantly higher in the control group. Moreover, the tocilizumab, corticosteroids use within 24 h of ICU admission, and the concomitant use of nephrotoxic medications during ICU stay were similar between the groups (Additional file 1: Table e1).

**Table 1:** Regression analysis for the outcomes after PS matching

	Outcomes			Hazard ratio (HR) (95%CI)	*P*-value$
	Control	Zinc	*P*-value^^		
In-hospital mortality, *n* (%)^§^	32 (40.0)	23 (28.4)	0.12	0.64 (0.37, 1.10)	0.11
30-day mortality, *n* (%)^§^	31 (38.8)	19 (23.2)	0.03	0.52 (0.29, 0.92)	0.03

			*P*-value^	beta coefficient (estimates) (95%CI)	*P*-value$*
Ventilator free days, Median (Q1, Q3)^§^	0.0 (0.00, 26.00)	20.0 (0.00, 28.00)	0.02	0.33 (− 0.21, 0.87)	0.22
ICU Length of Stay (Days), Median (Q1, Q3)^&^	8.0 (5.00, 15.00)	10.0 (6.00, 15.00)	0.28	0.10 (− 0.16, 0.36)	0.46
Hospital Length of Stay (Days), Median (Q1, Q3)^&^	16.0 (10.00, 28.00)	17.0 (12.00, 29.00)	0.61	0.03 (− 0.21, 0.27)	0.79

^§^Denominator of the percentage is the total number of patients

^&^Denominator is patients who survived

^^Chi-square test is used to calculate the *P*-value

^Wilcoxon rank sum test is used to calculate the *P*-value

^$^Cox proportional hazards regression analysis is used to calculate hazard ratio (HR) and *p*-value

^$*^Generalized linear model is used to calculate beta coefficient (estimates) and *p*-value

### 30-Day and in-hospital mortality

**Fig. 2:**
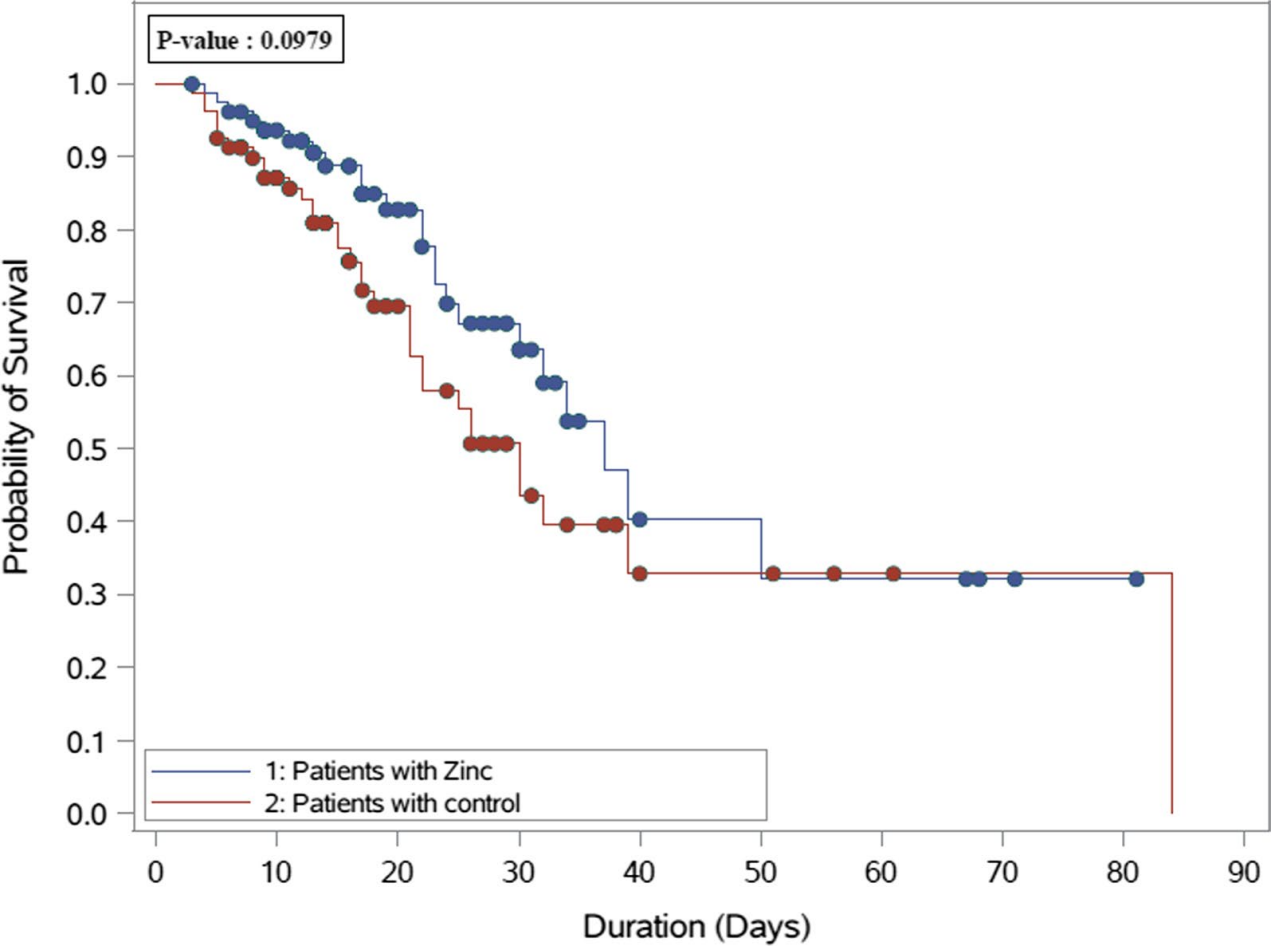
Overall survival plot during hospital stay after PS matching comparing patient who received zinc as adjunctive therapy (82 patients) versus control group (82 patients)

In crude analysis, 19 patients (23.2%) died within 30 days among the zinc group, compared with 31 patients (38.8%) in the control group (*P* = 0.03). At multivariable Cox proportional hazards regression analyses, the 30-day mortality was lower in patients who received zinc sulfate (HR 0.52 CI 0.29, 0.92; *p* = 0.03). On the other hand, the in-hospital mortality was similar between the two groups (HR 0.64 CI 0.37, 1.10; *p* = 0.11) (Table 1). The overall survival probabilities using Kaplan–Meier (KM) plots were not statistically different during hospital stay among patients who received zinc after propensity score-matched (*P* = 0.0979) (Fig. 2).

### Ventilator free days (VFDs) and length of stay (LOS)

Patients who received zinc have a longer median of VFDs than patients who did not (twenty versus zero days;* P* = 0.02). However, it was not statistically significant in the regression analysis (Beta coefficient 0.33 CI − 0.21, 0.87; *p* = 0.22). There were no statistically significant differences in ICU and hospital LOS between the two groups after using PS matching (Table 1).

### Complications during ICU stay

The acute kidney injury occurred in 10 patients who received zinc, compared with 25 patients in the control group (15.4% vs. 31.3%; *P* = 0.02). However, in the logistic regression analysis, it did not reach the statistical significance (OR (95%CI) 0.46 (0.19, 1.06), *p* = 0.07). The concomitant use of nephrotoxic medications was assessed and not statistically significant before and after PS matching between the two groups. Moreover, among the zinc group, only two patients (2.5%) have an acute liver injury versus seven patients (8.8%) in the control group. However, it was not statistically significant (OR (95%CI) 0.24 (0.05, 1.26), *p* = 0.09). No significant differences were observed in thrombosis/infraction (OR (95%CI) 0.46 (0.11, 1.98), *p* = 0.29) or respiratory failure requiring MV (OR (95%CI) 0.98 (0.31, 3.14), *p* = 0.98) (Table 2).

**Table 2:** Regression analysis for ICU complication (s) after PS matching

Outcomes	Crude analysis		*P*-value^^	Odds Ratio (OR) (95%CI)	*P*-value*^
	Control	Zinc			
Acute kidney injury, *n* (%)^§^	25 ( 31.3)	10 ( 15.4)	0.02^^	0.46 (0.19, 1.06)	0.07
Liver injury, *n* (%)^§^	7 ( 8.8)	2 ( 2.5)	0.08**	0.24 (0.05, 1.26)	0.09
Thrombosis/infraction, *n* (%)^§^	7 ( 8.8)	3 ( 3.8)	0.19**	0.46 (0.11, 1.98)	0.29
Respiratory failure requiring MV, *n* (%)^$^*	9/26 (34.6)	9/27 (33.3)	0.92^^	0.98 (0.31, 3.14)	0.98

^§^After zinc initiation

^$*^Denominator of the percentage is non-mechanically ventilated patients with 24 h of ICU admission

^^Chi-square /**Fisher Exact test is used to calculate the *P*-value

^*^^Multivariable logistic regression analysis is used to calculate Odds ratio and *p*-value

### Follow-up inflammatory surrogate markers

Comparing the effect of zinc on the COVID-19 inflammatory markers, patients who received zinc as new initiation in the ICU stay have a lower d-dimer (Beta coefficient − 0.80 CI − 1.28, − 0.32; *p* =  < 0.001) and fibrinogen levels (Beta coefficient − 0.99 CI − 1.51, − 0.48; *p* = 0.0002) as a follow-up surrogate marker than the control group as shown in Table 3.

**Table 3:** COVID-19 Inflammatory surrogate markers: peak levels follow-up

	Surrogate markers*		*P*-value^	Beta coefficient (estimates) (95%CI)	*P*-value$*
	Control	Zinc			
Ferritin level (ug/l), Median (Q1, Q3)^§^	891.9 (350.0, 2476.0)	941.1 (511.0, 2344.0)	0.69	0.21 (− 0.18, 0.60)	0.29
D-dimer level (mg/l), Mean (SD)^§^	15.0 (36.8)	6.7 (9.2)	0.75	− 0.80 (− 1.28, − 0.32)	0.001
Fibrinogen Level (gm/l), Median (Q1, Q3)^§^	5.5 (3.39, 7.18)	4.8 (3.95, 6.65)	0.31	− 0.99 (− 1.51, − 0.48)	0.0002
C-reactive protein (mg/l), Mean (SD)^§^	184.2 (148.1)	174.0 (124.3)	0.92	− 0.05 (− 0.36, 0.27)	0.77

^§^Denominator is the total number of patients

^Wilcoxon rank sum test is used to calculate the P-value

^$*^Generalized linear model is used to calculate estimates and p-value

^*^All levels represent peak levels for each one of the biomarkers

## Discussion

In this two-center, retrospective, propensity score matching study, zinc use as adjunctive therapy was associated with significantly lower 30-day mortality in critically ill patients with COVID-19; but the in-hospital mortality did not reach to a statistically significant difference. The overall survival probabilities were not statistically different during hospital stay between the two groups after propensity score. In addition, there was no significant difference in the MV duration, ICU and hospital LOS between the patients who used zinc and those who did not. Instead, patients who received zinc had lower odds of developing AKI; however. this finding did not reach statistical significance.

We have observed survival benefits with zinc supplementation within 30 days of hospital stay in critically ill COVID-19 patients. A retrospective study have shown that using zinc in combination with hydroxychloroquine and azithromycin in hospitalized patients with COVID-19 reduces mortality, ICU admission, and MV needs than patients who did not receive zinc[23]. However, a subgroup analysis in the same study of severely ill patients with COVID-19 found that zinc was not associated with a significant reduction in terms of in-hospital mortality [23]. A recent meta-analysis compared the outcomes of hospitalized patients receiving zinc supplementation with standard care [24]. This meta-analysis concluded that zinc supplementation did not have any beneficial impact on hospital mortality in COVID-19 patients [24]. It is noteworthy that the included studies in the meta-analysis were not critically ill patients, where the disease severity and low pre-admission nutritional status could justify the survival benefit observed in our analysis.

The pre-admission nutritional status is an important indicator for disease severity and might impact the COVID-19 patient's survival. A higher risk of mortality and longer stay in hospital was reported in critically ill COVID-19 patients with higher Nutritional Risk Screening (NRS) 2002 [25]. We assessed our patients' nutritional status using the NUTRIC score, which was not significantly different among the groups after propensity score matching. It worth to mention, that Saudi population has a low intake of some micronutrients (such as zinc and selenium) which might have an impact on the preadmission nutritional status and the disease progression [26]. In agreement with our explanation, a recent study has evaluated the impact of preadmission zinc levels in non-critically ill COVID-19 patients and showed that patients with hypozincemia have a higher hospitalization rate due to respiratory complications within ten days [27]. Thus, the impact of hypozincemia on the disease progression and the clinical outcomes still worth further investigation in critically ill COVID-19 patients.

Several reports showed that elevated inflammatory surrogate markers such as d-dimer and fibrinogen levels had been linked with a higher mortality rate in critically ill COVID-19 patients [4, 28, 30]. Zinc effect on COVID-19 surrogate markers was not well studied. In our study, patients who received zinc supplementation had lower follow-up d-dimer and fibrinogen levels during their stay. This may play a role in COVID-19 disease progression, which might be translated to the mortality benefit seen in our study.

There were no statistically significant differences in the ICU LOS and hospital LOS between the two groups after using PS matching. Our results are consistent with previous retrospective studies and randomized controlled trials (RCTs) showing that the concomitant use of zinc with hydroxychloroquine did not affect the ICU LOS, hospital LOS, or duration of MV [5, 22]. A systematic review of four RCTs conducted in non-COVID-19 critically ill patients found that zinc supplementation was not associated with significant differences in the duration of MV, ICU LOS, and hospital stay [18]. It is worth mentioning that most of these studies included patients with mild, and moderate  COVID-19 but not critically ill patients [5, 22].

In terms of the complications during ICU stay, zinc use was associated with a lower odd of acute kidney injury; however, did not reach statistical significance. We included the AKI status within 24 h of ICU admission in PS analysis; additionally, the concomitant use of nephrotoxic medications was assessed and it was not statistically significant after PS matching between the two groups. The exact mechanism and explanation for the reduced AKI risk observed in our cohort are unknown; zinc was thought to reduce renal injury incidence via its antioxidant effect in pre-clinical studies [25]. The zinc renal protective effect and the precise impact of zinc supplementation on renal function in critically ill COVID-19 patients are worth further investigation.

Currently, the available evidence about the use of zinc in critically ill COVID-19 patients is limited. Many of the previous reports investigated zinc use in either non-COVID-19 or non-critically ill patients. As we are writing this manuscript, there is an ongoing double-blinded RCT study investigating the use of high-dose zinc in critically ill patients with SARS-COV2 [29]. Compared to the published data, our study is one of the few studies that assessed the use of zinc in critically ill patients with COVID-19 with a propensity-score-matched group of patients.

Nonetheless, this study still has several limitations, such as the observational nature, small sample size, and the possibility of residual confounding may remain despite using propensity score matching. Additionally, our findings might be limited due to the administration of zinc via the enteral route, which might reduce the absorption in critically ill patients due to gastrointestinal ischemia and impaired intestinal flora. Even though zinc levels are challenging to measure accurately, this level might be valuable in determining the amount of zinc absorbed. The unavailability of zinc levels in this study might also limit our results. Furthermore, there was no defined treatment regimen, and the choice to initiate zinc was left to the discretion of the clinician. Given these limitations, the study's findings should not be used to guide clinical practice but to support the need for future RCTs to investigate the potential impact of zinc supplementation in critically ill patients with COVID-19.

## Conclusion

The use of zinc sulfate as adjunctive therapy in critically ill patients with COVID-19 may have survival benefits. Additionally, zinc use may have a protective effect on the kidneys. Further randomized clinical and interventional studies are needed to confirm our findings.

## Supplementary Information

**Additional file 1: Table e1** Patients Baseline characteristics before and after propensity-score matching.

## Data Availability

The datasets used and/or analyzed during the current study are available from corresponding author on reasonable request.

## Abbreviations

ICUs: Intensive care units
COVID-19: Coronavirus disease
MV: Mechanical ventilation
LOS: Length of Stay
APACHE II: Acute Physiology and Chronic Health Evaluation II
SOFA: Sequential Organ Failure Assessment
NUTRIC: Nutrition Risk in Critically ill
Q1, Q3: 1St interquartile and 3rd interquartile
eGFR: Glomerular filtration rate
AKI: Acute Kidney Injury
MV: Mechanical Ventilation
INR: International normalized ratio
aPTT: Activated partial thromboplastin time
C-RP: C-reactive protein
CPK: Creatine phosphokinase
PaO_2_/FiO_2_: Arterial oxygen tension/fraction of inspired oxygen
