# Supplementary material for: Evaluation of zinc sulfate as an adjunctive therapy in COVID-19 critically ill patients: a two center propensity-score matched study
Source: Crit Care. 2021 Oct 18;25:363. doi: 10.1186/s13054-021-03785-1 (PMC8522856; doi:10.1186/s13054-021-03785-1)
Supplement: Supplementary file 1 — Additional file 1: Table e1 Patients Baseline characteristics before and after propensity-score matching. [file 13054_2021_3785_MOESM1_ESM.docx]

**Additional file 1: Summary of Demography and Baseline characteristics**

|  | **Before propensity score (PS) matching** | | | | **After propensity score (PS) matching** | | | |
| --- | --- | --- | --- | --- | --- | --- | --- | --- |
|  | **Overall**  **(n=756)** | **Control (n=666)** | **Zinc**  **(n=90)** | **P-value** | **Overall (n=164)** | **Control (n=82)** | **Zinc (n=82)** | **P-value** |
| **Age (Years), Mean (SD)** | 60.8 (14.63) | 61.2 (14.7) | 58.1 (13.5) | 0.0603^ | 58.1 (15.2) | 58.0 (16.5) | 58.2 (13.9) | 0.9395* |
| **Gender – Male, n (%)** | 516 (70.9 ) | 448 (70) | 68 (77.3 ) | 0.1591^^ | 119 ( 73.5 ) | 54 ( 67.5 ) | 65 ( 79.3 ) | 0.0899^^ |
| **Weight (kg), Mean (SD)** | 80.6 (18.) | 79.9 (18.2) | 85.5 (19.9) | 0.0208^ | 82.0 (19.3) | 79.0 (18.1) | 85.0 (20.1) | 0.0701^ |
| **APACHE II score, Median (Q1,Q3)** | 15.0 (9, 25) | 15.0 (10, 25) | 11.0 (7, 14) | <.0001* | 11.0 (7 ,14.1) | 11.0 (7, 14) | 11.0 (7, 14) | 0.9745^ |
| **SOFA score, Median (Q1,Q3)** | 5.0 (3, 8) | 5.0 (3, 8) | 4.0 (2, 6) | 0.0002^ | 4.0 (2, 6.) | 4.0 (2, 7) | 4.0 (2, 6) | 0.5581^ |
| **NUTRIC score, Median (Q1,Q3)** | 4.0 (2, 6) | 4.0 (2, 6) | 2.0 (1, 4) | <.0001* | 3.0 (1, 4) | 3.0 (1, 4) | 2.0 (1, 4) | 0.7419^ |
| **NUTRIC score < 5, n (%)** | 434 ( 63.5 ) | 368 ( 61 ) | 66 ( 81.5 ) | 0.0003^^ | 134 ( 83.8 ) | 68 ( 86.1 ) | 66 ( 81.5 ) | 0.4309^^ |
| **NUTRIC score > 5, n (%)** | 250 ( 36.5 ) | 235 ( 39) | 15 ( 18.5 ) |  | 26 ( 16.3 ) | 11 ( 13.9 ) | 15 ( 18.5 ) |  |
| **Systemic corticosteroids use within 24 hours of ICU admission, n (%)** | 484 ( 66.2 ) | 425 ( 66.2 ) | 59 ( 66.3 ) | 0.9862^^ | 104 ( 64.2 ) | 51 ( 63.8 ) | 53 ( 64.6 ) | 0.9066^^ |
| **Tocilizumab use within 24 hours of ICU admission, n (%)** | 94 ( 12.9 ) | 80 ( 12.5 ) | 14 ( 15.7 ) | 0.3879^^ | 26 ( 16 ) | 13 ( 16.3 ) | 13 ( 15.9 ) | 0.9452^^ |
| **eGFR(ml/min/1.73m^2), Median (Q1,Q3)** | 73.0 (41, 9) | 70 (40, 96) | 83 (62, 103) | 0.0027^ | 83(60, 104) | 84.5 (58, 109.5) | 83. (62, 101) | 0.6112* |
| **AKI within 24 hours of ICU admission, n (%)** | 202 (28.7 ) | 188 (30.4 ) | 14 (16.3 ) | 0.0068^^ | 26 (16 ) | 12 (15.) | 14 (17.1 ) | 0.7193^^ |
| **MV within 24 hours of ICU admission , n (%)** | 538 (73.8 ) | 478 (74.7 ) | 60 (67.4 ) | 0.1438^^ | 109 (67.3 ) | 54 (67.5 ) | 55 (67.1 ) | 0.9538^^ |
| **Oxygenation Index (OI)** | 16.1 (8.6, 24.6) | 15.7 (8.4, 23.5) | 20.2 (11.9, 39.1) | 0.2380^ | 16.2 (8.4, 25.1) | 14.8 (8.4, 22.9) | 19.0 (9.61, 41.3) | 0.3845^ |
| **Lactic acid (mmol/l), Median (Q1,Q3)** | 1.7 (1.3, 2.5) | 1.7 (1.3, 2.5) | 1.7 (1.3, 2.1) | 0.3950^ | 1.8 (1.4, 2.4) | 2.0 (1.5, 2.5) | 1.7 (1.3, 2.1) | 0.0315^ |
| **Platelets count (10^9/l), Median (Q1,Q3)** | 245.5 (189.5, 319) | 245 (188.5, 319.5) | 251(198, 309.5) | 0.8381^ | 252(199.5, 322) | 248.5 (198, 332) | 252(204, 309) | 0.8111^ |
| **Total White blood cells (10^9/l), Median (Q1,Q3)** | 15.3 (10.9, 21.7) | 15.6 (11., 22.3) | 13.4 (9.3, 17.4) | 0.0051^ | 13.9 (10, 18.2) | 14.5 (10.3, 18.8) | 13.0 (9.3, 17.2) | 0.1797^ |
| **INR, Median (Q1,Q3)** | 1.1 (1., 1.2) | 1.1 (1., 1.2) | 1.1 (1, 1.1) | 0.0001^ | 1.1 (1., 1.1) | 1.1 (1., 1.) | 1.1 (1, 1.1) | 0.0881^ |
| **aPTT (Seconds), Median (Q1,Q3)** | 30.2 (27, 33.8) | 30.5 (27., 34.1) | 29.0 (25.1, 32.2) | 0.0051^ | 29.6 (26, 32.8) | 30.1 (26.7, 33.3) | 29 (25.3, 32.2) | 0.0996^ |
| **Total bilirubin (umol/l), Median (Q1,Q3)** | 10(7., 14) | 10.0 (7, 14.1) | 10(6.7, 13) | 0.6860^ | 9.8 (7, 14) | 9.2 (7.5, 15.3) | 10.0 (6.8, 13) | 0.8107^ |
| **Albumin Baseline (gm/l), Median (Q1,Q3)** | 33.2 (29.5, 37) | 34 (29.5, 37) | 32.5 (29.5, 35) | 0.0215^ | 32.(29, 36) | 32 (28, 36) | 33(30, 35) | 0.8604* |
| **CPK baseline (U/l), Median (Q1,Q3)** | 189.5 (77, 476.5) | 184 (76, 467) | 268(87, 557) | 0.1839^ | 235.5 (87, 554.) | 190(77, 41) | 287(107, 56) | 0.1104^ |
| **C-RP baseline (mg/l), Median (Q1,Q3)** | 150(85.6, 223) | 150.5 (86.5, 224.5) | 142 (75, 205) | 0.4177^ | 141 (79, 20) | 140.5 (84, 220.5) | 142 (77, 201) | 0.7690^ |
| **Procalcitonin (ng/ml), Median (Q1,Q3)** | 0.4 (0.2, 1.5) | 0.4 (0.2, 1.5) | 0.2 (0.1, 0.7) | 0.0112^ | 0.2 (0.1, 0.8) | 0.3 (0.2, 1.5) | 0.2 (0.1, 0.7) | 0.2851^ |
| **Fibrinogen Level (gm/l), Median (Q1,Q3)** | 5.5 (3.8, 7.2) | 5.5 (3.7, 7.3) | 5.8 (4.7, 7.2) | 0.2214^ | 5.9 (4.7, 7.3) | 6.2 (4.9, 7.3) | 5.7 (4.7, 7.1) | 0.6994^ |
| **D-dimer Level (mg/l), Median (Q1,Q3)** | 1.5 (0.8, 3.7) | 1.5 (0.8, 3.8) | 1.1 (0.7, 3.1) | 0.1336^ | 1.1 (0.6, 3.3) | 1.2 (0.6, 4) | 1.1 (0.7, 3.2) | 0.9244^ |
| **Ferritin Level (ug/l), Median (Q1,Q3)** | 836.7 (400, 1919) | 843.5 (400.5, 1917.5) | 802.8 (384.3, 1964) | 0.8391^ | 890.2 (384.3, 2115) | 913.1 (343.2, 2265.5) | 857.9 (414.2, 2106) | 0.8429^ |
| **Blood glucose level (mmol/l), Median (Q1,Q3)** | 11.5 (7.8, 16.1) | 11.7 (8, 16.2) | 10.4 (6.3, 15.9) | 0.0933^ | 10.7 (7.3, 16.3) | 11.6 (7.7, 17.4) | 10.3 (6.3, 15.8) | 0.1405^ |
| **PaO2/FiO2 ratio within 24 hours of admission, Median (Q1,Q3)** | 85.3 (60.9, 146.2) | 84.5 (60.4, 142.9) | 90.8 (65.8, 180.5) | 0.1516^ | 88.6 (58.3, 157.4) | 82.2 (58, 136.8) | 90.7 (65., 180.50) | 0.1523^ |
| **Respiratory Rate (breath per minute), Median (Q1,Q3)** | 28 (24, 34) | 28 (23, 34) | 30 (25, 35) | 0.0060^ | 30(23, 35) | 28 (22, 33) | 30.(25, 35) | 0.0241^ |
| **Maximum body temperature (Celsius), Median (Q1,Q3)** | 37.5 (37, 38.2) | 37.5 (37, 38.2) | 37.3 (37.1, 38) | 0.3558^ | 37.4 (37.1, 38.1) | 37.5 (37, 38.2) | 37.3 (37.1, 38) | 0.3226^ |
| **Prone position status, n (%)** | 197 ( 27.9 ) | 175 ( 28.2 ) | 22 ( 25.3 ) | 0.5670^^ | 47 29.6) | 24 ( 30.8) | 23 ( 28.4) | 0.7429^^ |
| **Patient received nephrotoxic drugs/material during ICU stay, n (%)** *$ | 598 ( 82.7 ) | 524 ( 82.6 ) | 74 ( 83.1 ) | 0.9077^^ | 134 ( 82.7 ) | 67 ( 83.8 ) | 67 ( 81.7 ) | 0.7310^^ |
| **Pharmacological DVT prophylaxis use during ICU stay, n (%)** | 627 ( 87.4 ) | 540 ( 86) | 87 ( 97.8 ) | 0.0017^^ | 155 ( 95.7 ) | 75 ( 93.8 ) | 80 ( 97.6 ) | 0.2330** |
| **Comorbidity, n (%)** |  |  |  |  |  |  |  |  |
| Atrial fibrillation (A Fib) , n (%) | 19 ( 2.6 ) | 18 ( 2.8 ) | 1 ( 1.1 ) | 0.3505** | 4 ( 2.5 ) | 3 ( 3.8 ) | 1 ( 1.2 ) | 0.2994** |
| Heart Failure, n (%) | 67 ( 9.2 ) | 62 ( 9.7 ) | 5 ( 5.6 ) | 0.2158^^ | 10 ( 6.2 ) | 5 ( 6.3 ) | 5 ( 6.1 ) | 0.9678** |
| Hypertension, n (%) | 415 ( 56.8 ) | 366 ( 57.0 ) | 49 (55.1 ) | 0.7274^^ | 86 ( 53.1 ) | 39 ( 48.8 ) | 47 (57.3 ) | 0.2747^^ |
| Diabetes mellitus , n (%) | 439 (60.1 ) | 388 ( 60.4 ) | 51 (57.3 ) | 0.5717^^ | 89 ( 54.9 ) | 42 ( 52.5 ) | 47 ( 57.3 ) | 0.5378^^ |
| Dyslipidemia, n (%) | 152 ( 20.8 ) | 131 ( 20.4 ) | 21 23.6 ) | 0.4870^^ | 33 ( 20.4 ) | 15 ( 18.8 ) | 18 ( 22.0 ) | 0.6130^^ |
| Ischemic heart disease (IHD) , n (%) | 55 ( 7.5 ) | 48 ( 7.5 ) | 7 ( 7.9 ) | 0.8964^^ | 10 ( 6.2 ) | 4 ( 5.0 ) | 6 ( 7.3 ) | 0.5401** |
| Chronic kidney disease (CKD) , n (%) | 92 ( 12.6 ) | 84 ( 13.1 ) | 8 ( 9.0 ) | 0.2750^^ | 13 ( 8.0 ) | 6 ( 7.5 ) | 7 ( 8.5 ) | 0.8082^^ |
| Cancer (any type) , n (%) | 23 ( 3.1 ) | 22 ( 3.4 ) | 1 ( 1.1 ) | 0.2434** | 4 ( 2.5 ) | 3 ( 3.8 ) | 1 ( 1.2 ) | 0.2994** |
| Deep vein thrombosis (DVT), n (%) | 7 ( 1.0 ) | 6 ( 0.9 ) | 1 ( 1.1 ) | 0.8638** | 1 ( 0.6 ) | 0 ( 0.0 ) | 1 ( 1.2 ) | 0.3218** |
| Pulmonary embolism (PE), n (%) | 6 ( 0.8 ) | 5 ( 0.8 ) | 1 ( 1.1 ) | 0.7355** | 2 ( 1.2 ) | 1 ( 1.3 ) | 1 ( 1.2 ) | 0.9860** |
| Liver disease (any type) , n (%) | 16 ( 2.2 ) | 12 ( 1.9 ) | 4 ( 4.5 ) | 0.1127** | 4 ( 2.5 ) | 0 ( 0.0 ) | 4 ( 4.9 ) | 0.0455** |
| Stroke, n (%) | 39 ( 5.3 ) | 37 ( 5.8 ) | 2 ( 2.2 ) | 0.1666** | 6 ( 3.7 ) | 4 ( 5.0 ) | 2 ( 2.4 ) | 0.3882** |
| *T Test / ^ Wilcoxon rank sum test is used to calculate the P-value.  ^^ Chi square/ ** Fisher’s Exact teat is used to calculate P-value.  *$Nephrotoxic medications/ material included IV Vancomycin, Gentamicin, Amikacin, Contrast, Colistin, Furosemide, and/or Sulfamethoxazole/trimethoprim | | | | | | | | |
